# Bilayer of Indium Phthalocyanine Molecules on Pb(001): Structure and Spin States

**DOI:** 10.1021/acs.jpclett.6c02063

**Published:** 2026-08-27

**Authors:** Niklas Ide, Arnab Banerjee, Alexander Weismann, Roberto Robles, Nicolás Lorente, Richard Berndt

**Affiliations:** † Institut für Experimentelle und Angewandte Physik, Christian-Albrechts-Universität, 24098 Kiel, Germany; ‡ Centro de Física de Materiales CFM/MPC (CSIC−UPV/EHU), 20018 Donostia−San Sebastián, Spain; § Donostia International Physics Center (DIPC), 20018 Donostia−San Sebastián, Spain

## Abstract

Bilayers of the nonplanar complex indium phthalocyanine (InPc) were studied on superconducting Pb(001) using scanning tunneling microscopy and density functional theory calculations. The structure of the bilayer is drastically different from that of a single layer in terms of the unit cell, the azimuthal orientations of the molecules, and the position of the In ion above (In↑Pc) or below (In↓Pc) the macrocycle. Intriguingly, the presence of the second layer appears to induce a geometric transition of the first layer. Moreover, a Yu–Shiba–Rusinov state reflecting a localized spin is observed above all molecules of the bilayer.

## Graphical abstract

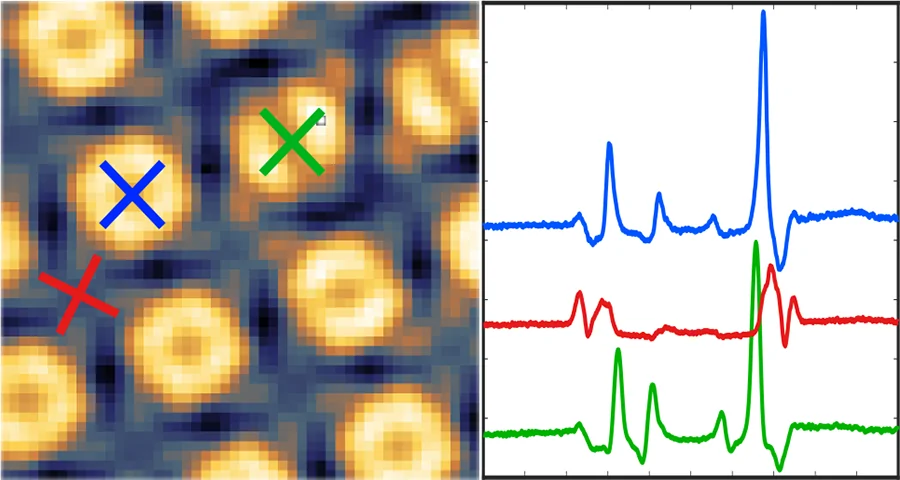

Many organic electronic devices rely on the interfaces between organic thin films and metal electrodes, and consequently, the electronic structure of such interfaces has been widely investigated. While electron spectroscopies have been used to study the relevant molecular energy levels,^1–4^ scanning tunneling microscopy (STM) is particularly helpful because it resolves the structure right at the interface and provides some information on the electronic states as well. Compared to the large number of investigations of single molecular layers, less STM data are available for bilayers and larger thicknesses. This is unfortunate because it should not be expected that the properties of bilayers can be extrapolated from the monolayer results. For example, in bilayers, the first molecular layer was used to partially electronically decouple the molecules of the second layer from the substrate. This served to achieve STM-induced light emission from perylenetetracarboxylic dianhydride molecules,^5^ led to enhanced vibrational excitation of iron phthalocyanine^6^ and tin phthalocyanine (SnPc),^7^ enabled a reversible geometric switching of the conformation of SnPc,^8^ and resulted in highly resolved orbital images of CoPc.^9^ The layer-resolved electronic properties have also been directly addressed.^10,11^ STM studies of structural aspects of bilayers have been reviewed in ref 12, and further studies have been reported.^13^ Spatially averaging techniques, like normal-incidence X-ray standing waves, have also been used to investigate the structure of heterobilayers.^14,15^

The spatially resolved magnetic properties of molecular bilayers have received little attention so far.^16^ Here, we address this aspect using indium phthalocyanine (InPc) on Pb(001). The adsorption to a metal substrate can induce a magnetic moment in this case, similar to observations from a few other phthalocyanines.^17–20^ Magnetic impurities on a superconducting surface can induce Yu–Shiba–Rusinov (YSR) states via the exchange interaction of the impurity spin and the substrate Cooper pairs.^21–25^ In spectra of the differential conductance d*I*/d*V*, these states can be resolved as peaks inside the superconducting gap. Therefore, these states are convenient sensors of localized magnetic moments in molecules if the spin–substrate interaction is large enough.^26–33^

InPc is a nonplanar molecule, and consequently, two conformations denoted In↑Pc and In↓Pc are observed on a lead substrate. The arrows indicate the position of the In ion above the macrocycle or between the macrocycle and the substrate. We find that the bilayer adopts a structure that drastically differs from the structure of a single layer. In contrast to the simplistic expectation of the second layer growing on top of the previously formed first layer, the second layer modifies the first layer by converting the molecules from In↑ to In↓. A localized spin is observed above all molecules of the bilayer, while only In↑Pc molecules carry a spin in the single layer.

## Geometric Structure

Figure 1a presents an overview of a Pb(001) surface after the deposition of a submonolayer amount of InPc. The image shows several Pb terraces within an area of (200 nm)^2^. Some isolated In↓Pc molecules can be observed on these terraces, while the majority of InPc molecules aggregate into islands along the step edges. Islands labeled 1 are single layers with a $\left(\sqrt{65}\times \sqrt{65}\right)$ *R*30° superstructure with four molecules per unit cell, as reported previously.^19^ In brief, one In↑Pc and three In↓Pc molecules occupy top, hollow, and bridge sites. While the isoindole lobes of the molecules in 4-fold hollow and top sites are parallel to ⟨1̅10⟩ substrate directions, the bridge-site molecules are azimuthally rotated by 10°.

**Figure 1:**
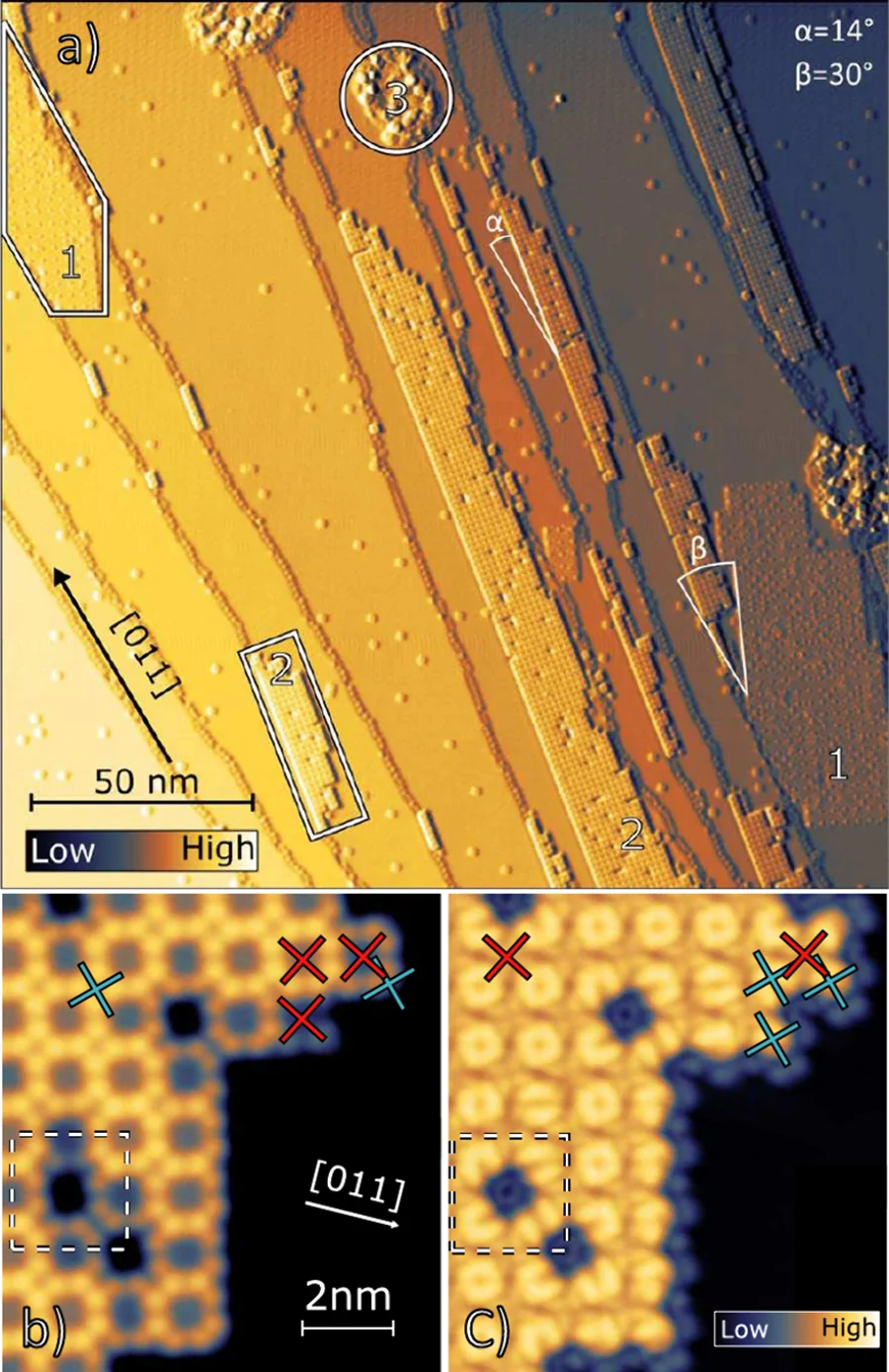
(a) Topograph of a submonolayer coverage of InPc on Pb(001) (*I* = 50 pA and *V* = 1 V). The data displayed are a weighed sum of the height *z* and its derivative d*z*/d*x* along the horizontal direction. The overview image shows multiple terraces of the Pb substrate separated by monatomic steps. Some monolayer and bilayer islands are labeled with 1 and 2, respectively. α (β) indicates the angle of a unit cell edge with respect to the substrate for bilayer (monolayer) islands. An example of a disordered cluster is labeled 3. (b and c) Topographs of an edge of a bilayer imaged at *V* = 1000 and 20 mV, respectively. Blue and red crosses indicate the isoindole lobes of molecules in the first and second layers, respectively.

In contrast, islands labeled 2 appear higher and also show a distinct superstructure. Closer inspection of these islands reveals a bilayer structure. Compared to the monolayer islands, bilayer islands are rotated by 16° and are predominantly observed along step edges. In bilayer islands, the second layer usually completely covers the first layer. A rare example of a bilayer with some uncovered first layer is shown in Figure S7 of the Supporting Information. Finally, some disordered clusters (3) are observed.

Figure 1b and c shows constant-current topographs of the edge of a bilayer island recorded at 1 V and 20 mV, respectively. First- and second-layer molecules are marked by blue and red crosses, respectively, which schematically represent the four lobes of the phthalocyanines. At low bias, a nearly doughnut-shaped, 4-fold symmetric maximum centered on top of first-layer molecules is observed. This feature results from the isoindole lobes of four neighboring molecules in the second layer. The contrast above a missing second-layer molecule (dashed square) supports this interpretation.

The lobes of the first-layer molecules are discernible at the island edge. The high-bias image shows two maxima on each isoindole lobe of the second-layer molecules (red crosses), while the first-layer molecules are invisible. These lobes are characteristic of the lowest unoccupied molecular orbital (LUMO) of Pc molecules.^17^ The azimuthal angles of the isoindole lobes relative to a ⟨110⟩ direction of the Pb lattice of the first- and second-layer molecules are 45° and 33°, respectively. The distance between nearest molecules in the second layer is 1.44 nm, and their centers are located above the hollow sites between the first-layer molecules. This corresponds to a $\left(\sqrt{17}\times \sqrt{17}\right)$ *R*14° superstructure with two molecules per unit cell, one in the first and one in the second layer.

## YSR States of Bilayers

Figure 2 shows d*I*/d*V* spectra measured above the center of a second-layer (red) and first-layer (blue) molecule at two different set points in panels a and b, respectively. Vertical lines display the voltage of the location of the peak in *V* = 1.85 mV.

**Figure 2:**
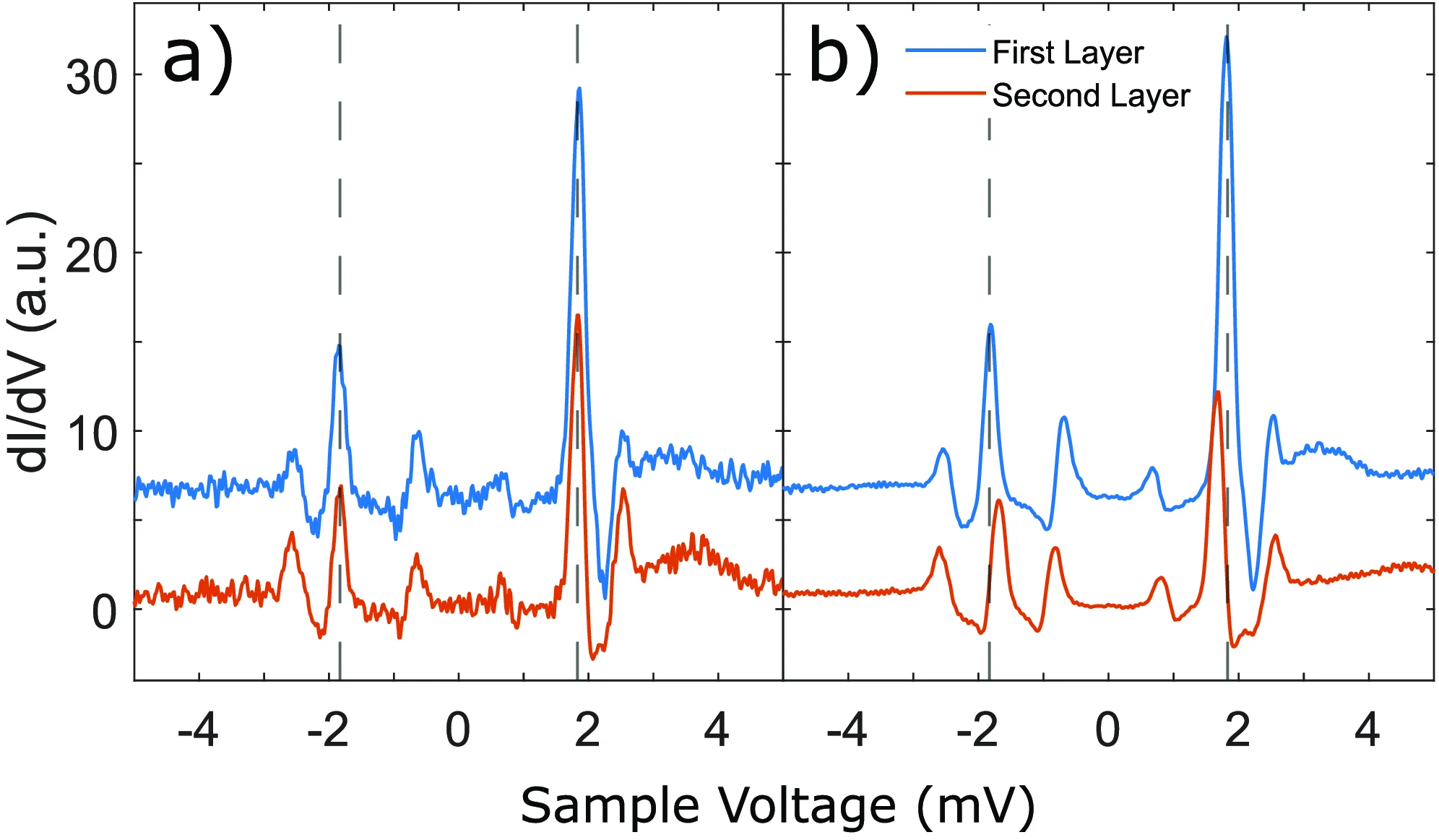
d*I*/d*V* spectra acquired above the centers of molecules in the second (red) and first (blue) layer at different tip heights. The tip position was frozen using *V* = 6 mV and *I* = 50 and 400 pA in panels a and b, respectively. Vertical lines indicate the YSR-state position determined in panel a, |*V*| = 1.85 mV. The peaks above first- and second-layer molecules occur at identical voltages in panel a. In panel b, at a lower tip height, the YSR peak is shifted when the tip is placed above second-layer molecules.

The superconductor coherence peaks appear at Δ/*e* = (Δ_T_ + Δ_S_)/*e* = 2.4 mV, where Δ_T/S_ denotes the superconducting gap of the tip/sample. At both positions, an in-gap state is observed at *eV* = ±(Δ_T_ + ϵ), and a copy of that state due to thermally excited carriers occurs at *eV* = ±(Δ_T_ – ϵ). At a large tip height, a single state is located at (Δ_T_ + ϵ)/*e* = 1.85 mV on both first- and second-layer molecules. Upon bringing the tip closer to the molecules, the in-gap state shifts inward to (Δ_T_ + ϵ)/*e* = 1.81 mV for first-layer molecules and to 1.71 mV for the second layer. We identify these peaks as being due to YSR states. It may be noted that non-magnetic scatterers can also give rise to sharp in-gap states on conventional superconductors under specific circumstances.^34^ These states have recently been reported from transmon qubits and quantum dots.^35–37^ However, this is not likely the case in the present study due to the magnetic moment derived from density functional theory (DFT) calculations.

To resolve further details, maps of d*I*/d*V* were measured at the YSR energies for the different set points. A topograph along with d*I*/d*V* maps of the same area are displayed in Figure 3. The positions and orientations of the first- and second-layer molecules are schematically indicated according to the results from Figure 1. The topograph (Figure 3a) shows the characteristic *C*_4_-symmetric rings (”doughnut”) around the centers of first-layer molecules. In addition, some rings (encircled by dashed lines) appear to be split by a nodal line (*C*_2_ symmetry). The origin of this symmetry breaking is unknown, and therefore, these areas are being ignored in the further analysis. In the d*I*/d*V* map (Figure 3b corresponding to the low set point YSR state *V* = 1.85 mV and *I* = 50 pA), we find intensity maxima above the centers of the first-layer molecules (blue dashed lobes), while the conductance exhibits minima above the lobes of the second-layer molecules (red dashed lobes). In addition, a sharp maximum is observed above second-layer molecules. Figure 3c and d shows the same area recorded with *V* = 1.71 and 1.81 mV, respectively, at *I* = 400 pA. At *V* = 1.71 mV, conductance maxima are observed on the centers and edges of the lobes of second-layer molecules, while minima are observed on top of first-layer molecules. At *V* = 1.81 mV, the conductance is maximal on the center of first-layer molecules, while minima are observed on second-layer molecules.

**Figure 3:**
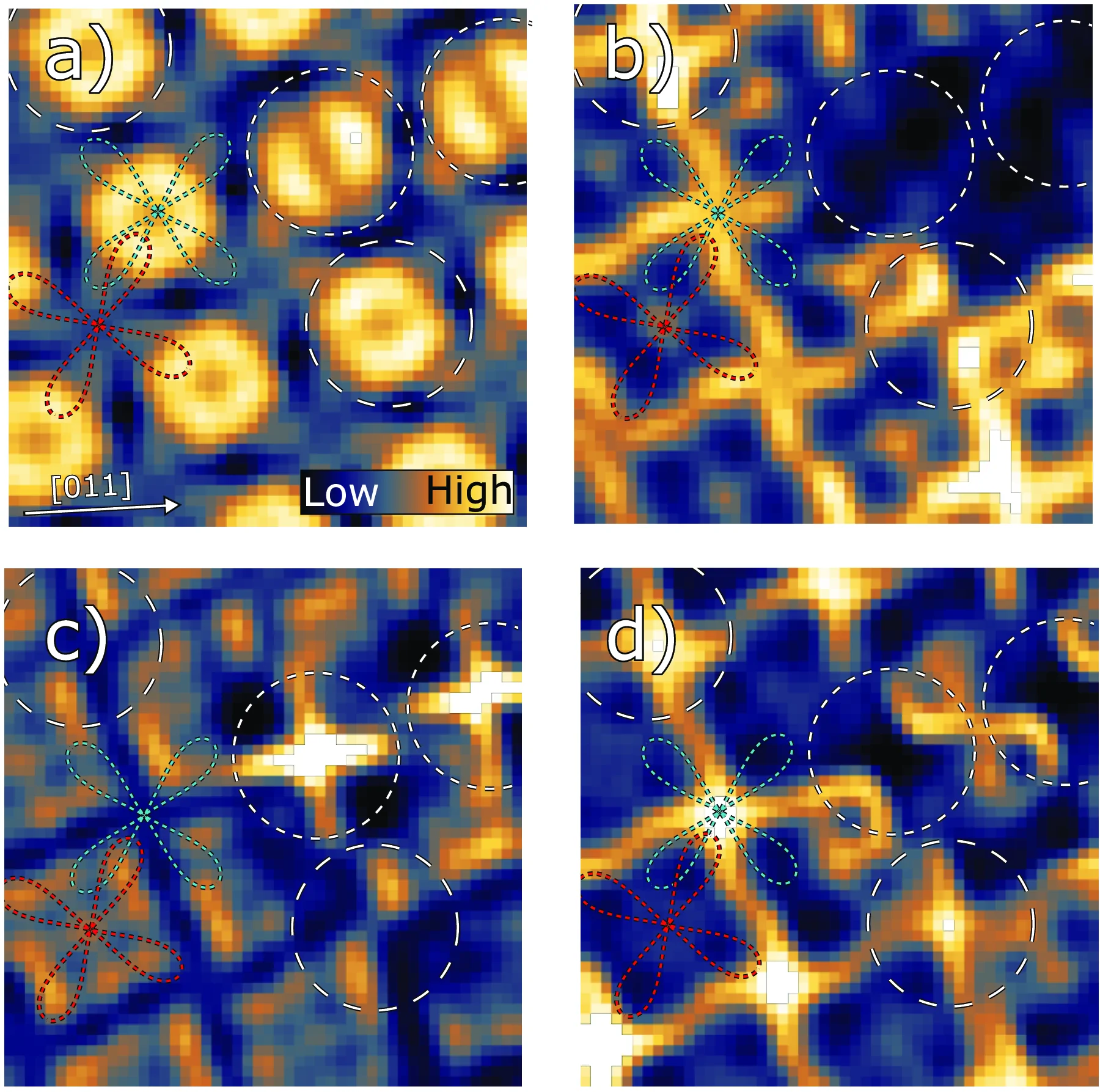
(a) Topograph of a (4 nm)^2^ area of a bilayer. Blue and red lines schematically indicate the clover leaf shape of the isoindole lobes of molecules in the first and second layers, respectively, determined from Figure 1. Dashed rings mark molecules with reduced symmetry (*C*_2_). The cause of the symmetry reduction is currently not known. (b–d) Maps of d*I*/d*V* from the same area, measured at a constant current. Panel b was measured at *V* = 1.85 mV and *I* = 50 pA corresponding to the YSR peak in Figure 2a. Panels c and d were obtained at *V* = 1.71 and 1.81 mV using *I* = 400 pA.

## DFT Modeling

To further elucidate the geometrical structure of the bilayer, we performed extensive DFT calculations. We used a $\left(\sqrt{17}\times \sqrt{17}\right)$ *R*14° unit cell, as determined from the experimental data. The unit cell contains two InPc molecules, one in the first layer and another in the second layer above the sites between the first-layer molecules. We calculated configurations with different adsorption sites and azimuthal angles of the molecules and also different positions of the In atom (In↑ or In↓). For all configurations, we simulated STM images at different voltages and determined the magnetic states (see the Supporting Information).

The structure that best matches the experimental observations is shown in Figure 4. It is the lowest energy structure found that also reproduces the experimental observation of a molecular spin moment. The overall lowest energy structure (Supporting Information, geometry I) is also compatible with the experimental observations, but it is not magnetic. The lack of magnetism could be due to the inability of DFT to correctly describe the electronic correlation of the relevant molecular orbitals.^38^ In any case, the particular details of the structure do not affect the main points of the discussion below, except, of course, the magnetic part.

**Figure 4:**
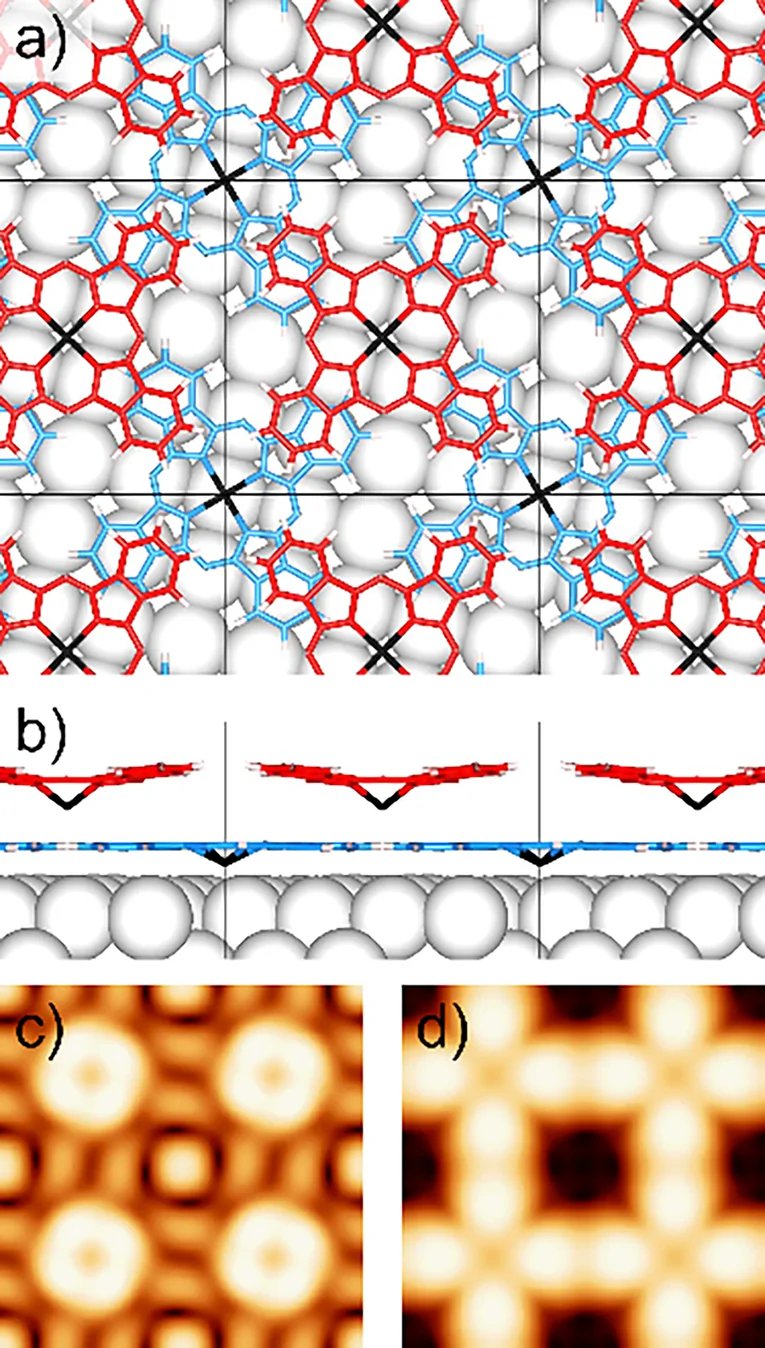
(a) Top and (b) side views of the structural model of the bilayer island. Molecules in the first (second) layer are represented by blue (red) sticks. White spheres represent Pb atoms. Black lines show the unit cell. Simulated constant-current STM images of the structure in panel a are shown for (c) *V* = 50 mV and (d) *V* = 1.0 V.

STM images are well-reproduced. In particular, we observe the doughnut pattern at low bias (Figure 4c), and the quite different image contrast at high bias is also present (Figure 4d).

The origin of the difference between low and high bias images can be traced back to an electronic state at ≈0.6 eV belonging to the molecule in the second layer. This state dominates the STM image for voltages above it, producing a similar shape for all computed configurations (see the Supporting Information). The molecules in the first layer are in direct contact with the Pb surface and acquire 0.71 electrons per molecule. The molecules in the second layer have only an indirect contact with the substrate, which decreases the hybridization and lowers the charge transfer to 0.30 electrons per molecule. Likewise, both molecules are magnetic. The molecules in the first layer carry a spin moment of −0.20 μ_B_ located mainly in the phthalocyanine cycle, while the molecules of the second layer have a spin moment of 0.46 μ_B_ located at the In ion and the nearby pyrrole N atoms (see the Supporting Information).

Unlike the structure of the monolayer,^19^ the bilayer is exclusively comprised of In↓Pc molecules. Actually, the low-voltage STM image calculated for the same structure with In↑Pc molecules in the first layer is qualitatively different (see the Supporting Information). In particular, the hole of the “doughnut” is absent in contrast to the experimental results (Figure 5). Moreover, the total energy is increased by 0.45 eV per molecule.

**Figure 5:**
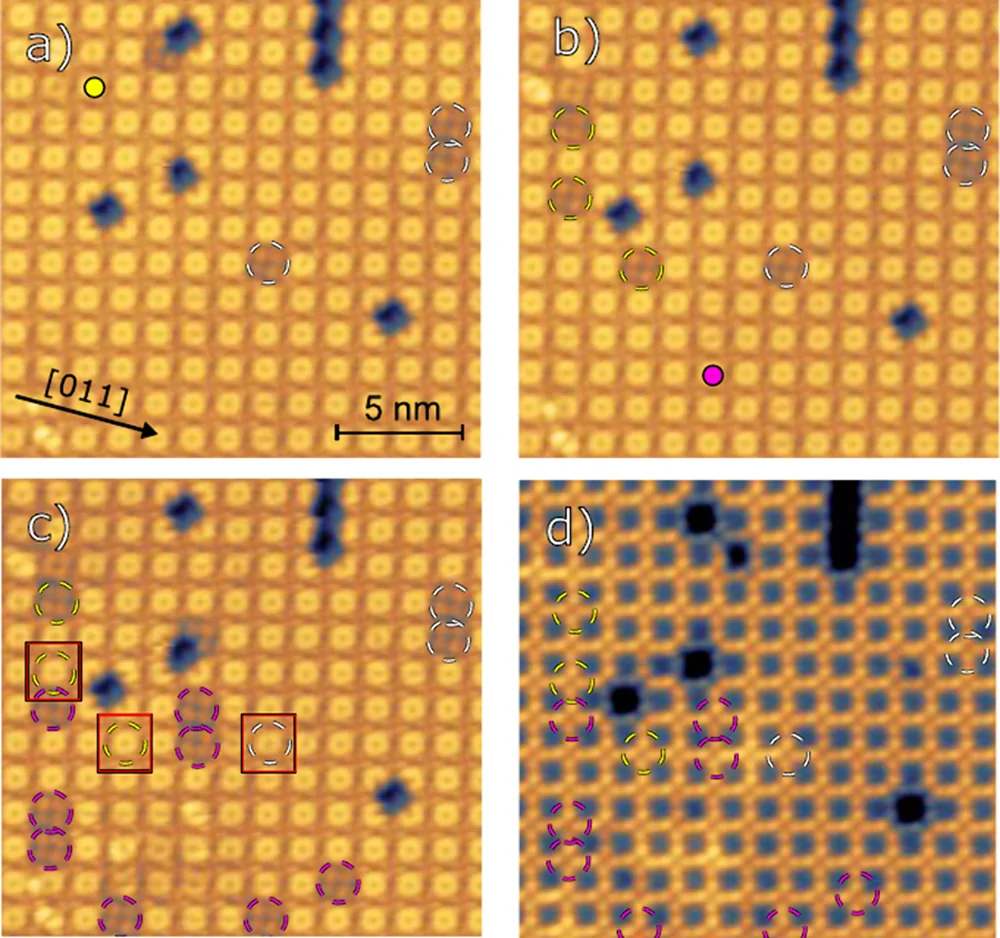
Inducing a geometrical transition in a bilayer island. (a) Initial state of a bilayer area with a few second-layer molecules missing. A yellow dot indicates the position where the voltage was raised to 3.5 V. (b) Image recorded subsequently. The positions indicated by yellow circles underwent a transition and appear lower. The manipulation procedure was then repeated at the positions marked by a pink dot. (c) Next image recorded. Several transitions (pink circles) were induced. Red squares indicate the positions of reversible transitions. Topographs a–c were measured with *V* = 20 mV. White circles indicate patterns that had been manipulated before recording the image in panel a. (d) Same surface status as in panel c, imaged at 1 V.

At first glance, the absence of In↑Pc molecules from the bilayer seems surprising because In↑ and In↓ conformers can be expected to land on the surface with equal probabilities. However, this absence can be understood by the decrease of the calculated barrier heights of the transition from In↑Pc to In↓Pc (see the Supporting Information). While the barrier height is 0.64 eV for the monolayer, it is reduced to 0.48 eV in the bilayer.

## STM-Induced Structural Transition

We previously showed that a transition from In↑Pc to In↓Pc may be induced on Pb(001) by centering the STM tip above an In↑Pc molecule and applying an elevated sample voltage.^19^ A related effect may be observed on the bilayer. Figure 5 shows an area of a bilayer after inducing multiple transitions by applying *V* = ±3.5 V. The topographs in panels a–c were measured at 20 mV, while the image in Figure 5d was obtained at 1 V. At low voltage, the bilayer displays the characteristic *C*_4_-symmetric doughnut patterns above first-layer molecules. Dark spots between the doughnut patterns are due to molecules missing from the second layer. Some *C*_4_-symmetric patterns (white circles) appear lower and were induced by prior manipulation experiments. A transition was induced by positioning the tip above the molecule in the first layer marked with a yellow dot in panel a and applying 3.5 V until a rapid change of the tunneling current was observed. The topograph measured subsequently (panel b) reveals a reduction of the apparent height on three first-layer molecules marked by yellow circles. A second manipulation was then made at the position of the pink circle in panel b. This again led to a transition of several molecules (panel c, pink circles). In addition, the patterns marked with a red square have been reverted to the original doughnut shape. At increased voltage (panel d), the image contrast is different and the isoindole lobes of the molecules in the second layer are resolved identically to Figure 1b.

The superstructures of the InPc monolayer and bilayer are distinctly different. The monolayer exhibits a $\left(\sqrt{65}\times \sqrt{65}\right)$ *R*30° superstructure,^19^ while the bilayer superstructure is $\left(\sqrt{17}\times \sqrt{17}\right)$ *R*14°. A related observation was previously made for SnPc molecules on Pb(001).^20^ They also assemble into two distinct superstructures depending on the position of the Sn ion above or below the macrocycle. Similar to a monolayer of the Sn↑Pc molecule, the molecules of the InPc bilayer are separated by a vector [4 1] in units of the nearest neighbor distance of Pb. All molecules in each molecular layer of the bilayer exhibit the same azimuthal orientation, whereas molecules in the monolayers of SnPc and InPc are rotated with respect to their neighbor molecules. This results in a smaller unit cell of the bilayer compared to the monolayer of SnPc (two molecules per unit cell) and InPc (four molecules per unit cell).

On the InPc monolayer, a YSR state is observed on top of In↑Pc but not on In↓Pc complexes. On the bilayer, we observe a YSR state from first-layer In↓Pc molecules. Moreover, a distinct YSR state is observed on the center of first- and second-layer molecules when the tip is brought closer to the sample (Figure 2). To further investigate this behavior, we recorded d*I*/d*V* spectra above the centers of first- and second-layer molecules at different tip heights. The YSR energies and peak height asymmetries χ = (*h* ^+^ – *h* ^–^)/(*h* ^+^ + *h* ^–^) are displayed in Figure 6. The YSR energies are reduced when the conductance is increased, leading to shifts Δϵ of 0.06 and 0.2 meV for first- and second-layer molecules, respectively. In addition, the asymmetry is reduced as the peaks shift toward the Fermi level. This effect is consistent with the results of refs 28 and 39 for MnPc and VOPc complexes.

**Figure 6:**
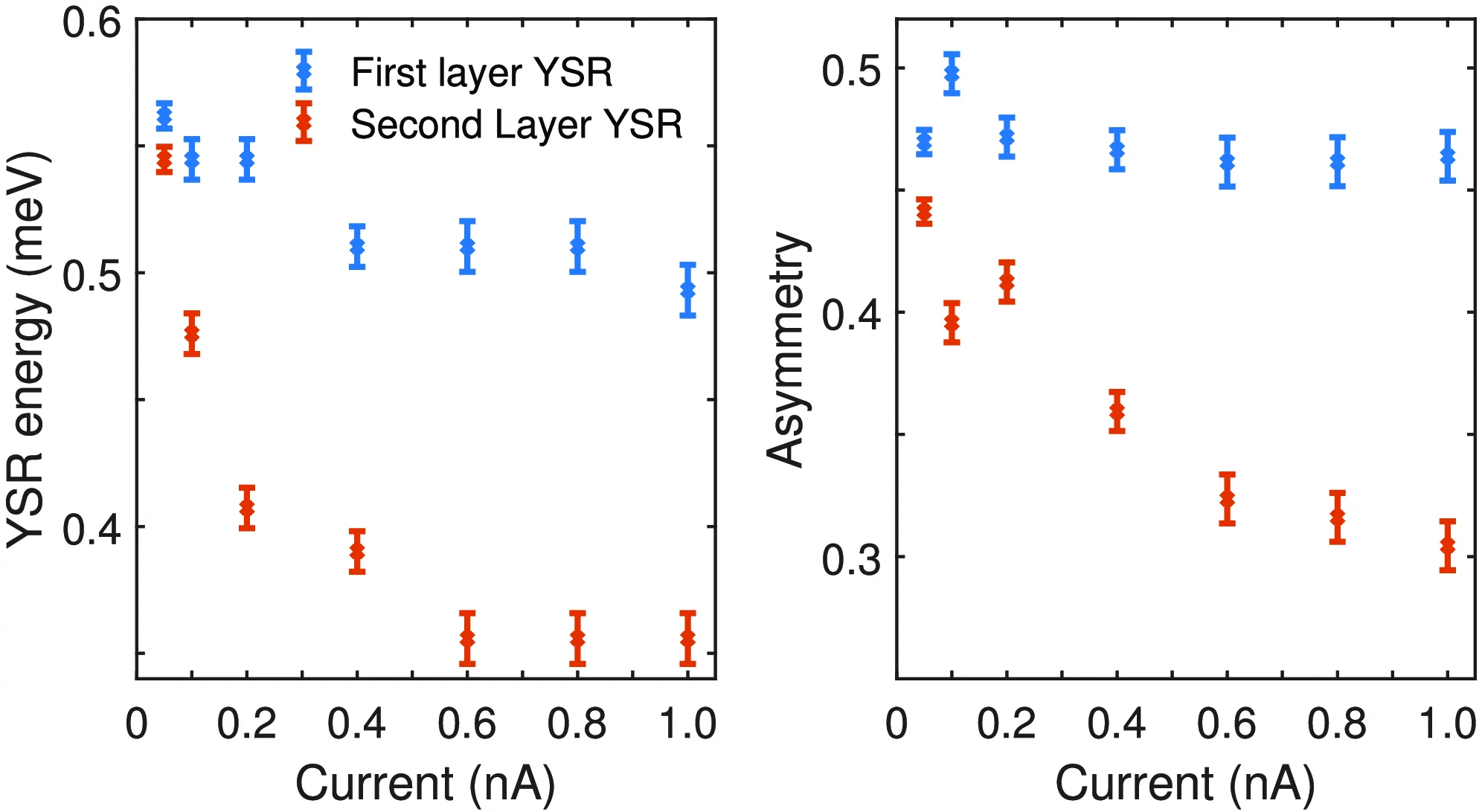
Energy of the YSR states as a function of the set point current *I* determined from d*I*/d*V* spectra taken above the centers of first-layer (blue) and second-layer (red) molecules. (Left) YSR energies were calculated according to ϵ = *eV* – Δ_T_. (Right) Corresponding asymmetry of the YSR peak according to χ = (*h* ^+^ – *h* ^–^)/(*h* ^+^ + *h* ^–^).

The fact that we do not observe both YSR peaks simultaneously raises the question of the spatial distribution of the two YSR peaks. Figure 7a shows a topograph of the same area as Figure 3. d*I*/d*V* spectra measured along the red line in panel a are shown in panel b, which connects the centers of two second-layer molecules and crosses a first-layer complex. Above the centers of the second-layer molecules (red arrows), the YSR state α lies particularly close to the Fermi level. Above the first-layer molecule (blue arrow), its energy is higher. Finally, above the lobes of the second-layer molecules (green arrows), two distinct YSR states are resolved.

**Figure 7:**
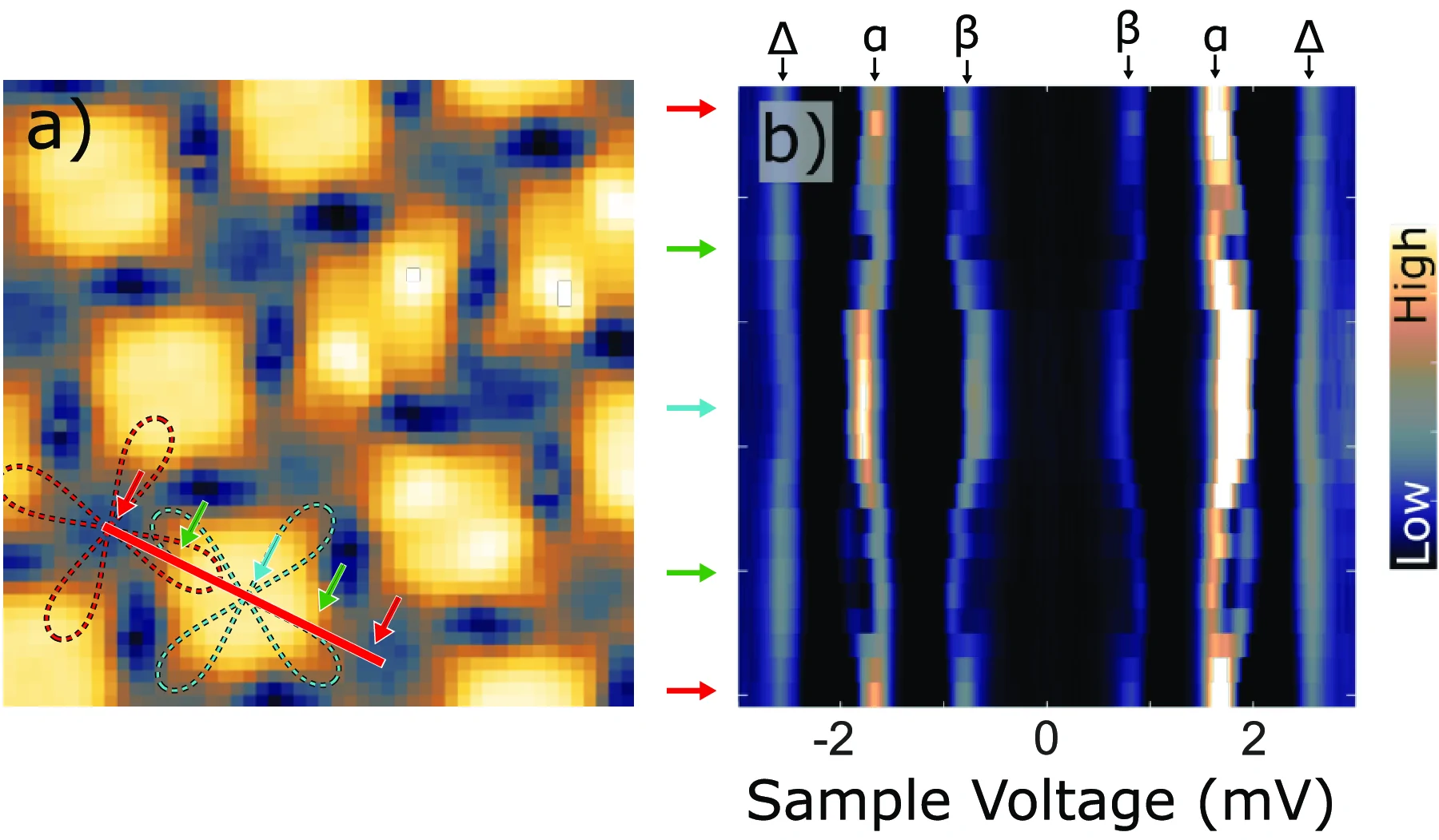
(a) Topograph of a (4 nm)^2^ area of a bilayer island, with *V* = 6 mV and *I* = 400 pA. Blue and red dashed lines indicate the clover leaf shape of first- and second-layer molecules, respectively. (b) d*I*/d*V* spectra obtained along the red line in panel a. Arrows indicate the positions of the recorded spectra. Blue and red lines indicate the positions of first- and second-layer molecules, respectively. The coherence peaks marked by Δ are resolved at *V* = 2.4 mV. YSR states are denoted α, and their copies due to thermally excited carriers are labeled β.

What is the origin of the distinct YSR states? Possibly, magnetic moments in the first and second layers lead to separate YSR states. However, the large separation of the second layer from the Pb substrate renders such an effect to be improbable.

For most Pc molecules, the spatial distribution of the YSR state matches the degenerate LUMO.^40^ Therefore, a more likely scenario is that the proximity of the tip breaks the degeneracy of the LUMO of the first-layer molecules and thus creates two separate YSR states at certain tip positions. At other positions, the tip may induce only a shift to lower energies. A related effect has been recently reported from a three-dimensional organometallic complex.^41^

Intriguingly, the bilayer islands are composed of a second layer that completely covers the first layer. We did not observe small patches of a second layer on top of a larger first layer, similar to the case of VOPc molecules on Bi(111).^13^

The reversible switching induced with STM (Figure 5) changes the contrast in low-bias STM images in the area above first-layer molecules. Simultaneously, high-bias images, which are mainly due to the LUMO of second-layer molecules, remain unaltered. We therefore discard a geometrical change of the configuration of the second layer as a possible origin of the switching. We rather favor a change in the first layer, as either a geometrical transition from In↓Pc to In↑Pc or an azimuthal rotation.

We first consider a In↓ to In↑ transition of a first-layer molecule. The voltage applied during a manipulation (3.5 V) provides sufficient electron energy to overcome the corresponding transition barrier height (0.967 eV; Figure S5a in the Supporting Information). However, the simulated low-bias STM image of the corresponding geometry with In↑Pc molecules in the first layer shows a conductance maximum located above the center of the first-layer molecule (Supporting Information, geometry VIII), which is not observed in the experimental data. We therefore discard this mechanism.

Next, we consider the rotation mechanism of a first-layer molecule, as illustrated in Figure 8. In order to do so, we increase the size of the unit cell to include twice the number of molecules. The left column shows the pristine geometry of Figure 4a, where the lobes of the first-layer molecules are oriented at 45° with respect to the Pb substrate. The simulated low-bias STM image exhibits the characteristic doughnut pattern at the center of the first-layer molecule, while the high-bias image displays two conductance maxima on each isoindole lobe of a second-layer molecule, in agreement with the experimental observations. In the geometry on the right column, the first-layer molecules have alternating orientations (35° and 45°). The low-bias STM image shows a “doughnut” pattern with different heights above the 35° and 45° molecules, while the high-bias image remains identical. These calculations show that the rotation of first-layer molecules can indeed reduce the apparent height in STM images, as observed in the experiments. Unfortunately, calculations for a much larger unit cell that could better mimic the rotation of a single first-layer molecule are computationally too costly. The proposed rotation mechanism should therefore be considered as a hypothesis rather than a settled fact.

**Figure 8:**
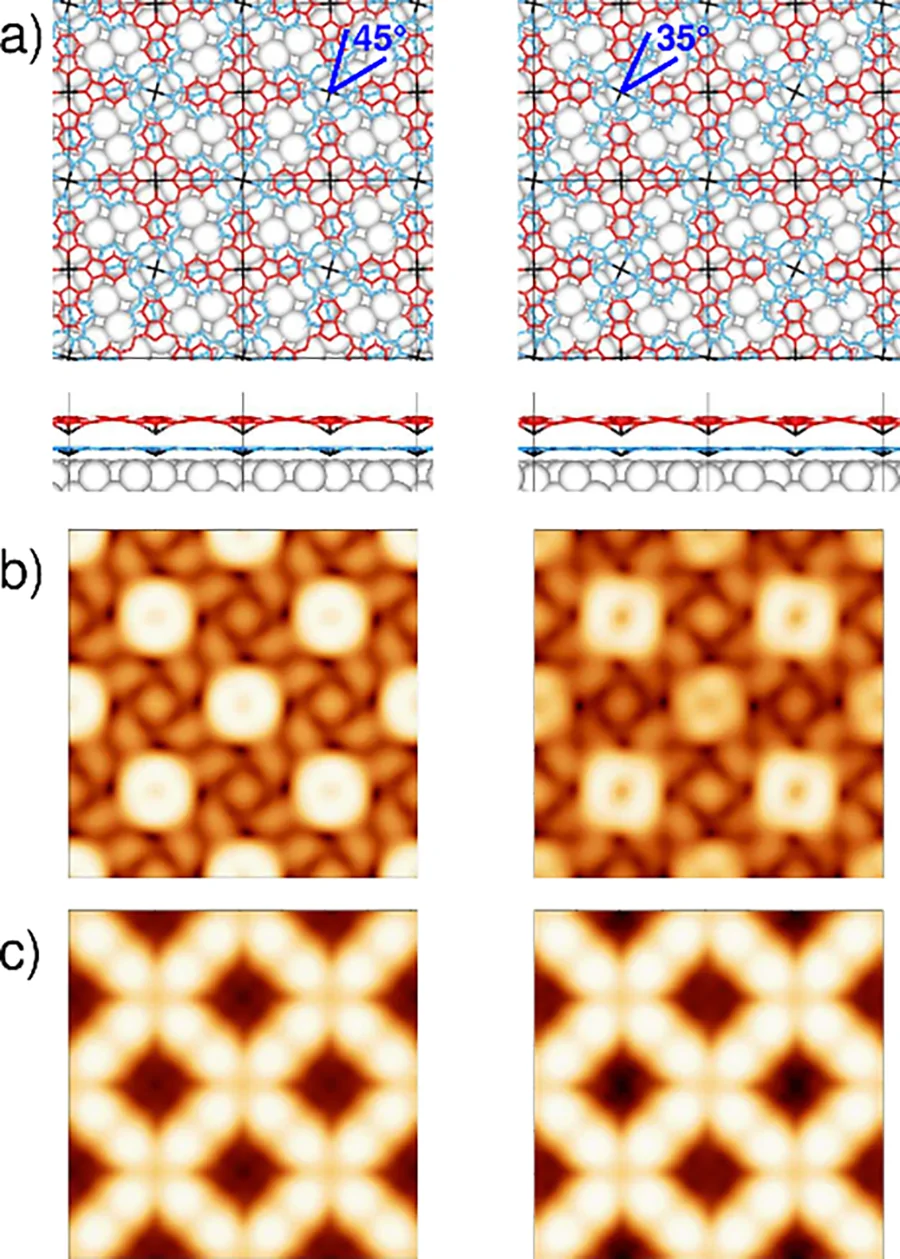
(a) Top and side views of the geometric structure of the bilayer in a doubled unit cell. The left column shows the same configuration as in Figure 4a, where the first-layer molecules exhibit an angle of 45° with respect to the Pb surface. The right column shows a configuration with two different orientations of first-layer molecules, 35° and 45°. Simulated constant-current STM images at (b) *V* = 50 mV and (c) *V* = 1 V, respectively. Note that this unit cell is rotated by 45° with respect to the one in Figure 4.

STM investigations of adsorbed molecules typically focus on (sub)­monolayer coverages, and data on thicker layers are rare. The results on a bilayer of InPc presented here show that naive expectations for the growth of multilayers can be misleading. The second InPc layer does not simply grow on top of the previously formed first layer. It rather modifies the structure of the first layer, where In↑Pc complexes are converted to In↓Pc. Furthermore, DFT calculations reveal an unexpected spin structure of the bilayer. Localized spins are found in all molecules of the bilayer, while only In↑Pc molecules carry a magnetic moment in the single layer. Spectral signatures of the magnetic moments are also observed in the experimental data. Taken together, the experimental and theoretical results suggest that exploring molecular spin effects beyond the monolayer regime may offer further surprises.

## Methods

### Experimental Details

A scanning tunneling microscope operating in ultrahigh vacuum at a temperature of 4.2 K was used. Pb(001) substrates were cleaned using Ar ion bombardment and annealing to approximately 230 °C. STM tips were cut from a Pb wire and ion-bombarded in ultrahigh vacuum. ClInPc molecules were sublimated from a Knudsen cell onto the Pb surface at ambient temperature, which leads to the loss of the Cl ion.^19^ For imaging, constant currents of 50 pA were chosen. d*I*/d*V* spectra were acquired by numerical differentiation of *I*(*V*) curves at the given positions. Maps of differential conductance were obtained by numerical differentiation of *I*(*V*) data measured on a grid of points. For manipulation experiments, the STM tip was placed above the center of a InPc molecule in the first layer, the tip position was frozen, and *V* was slowly increased. A sudden fluctuation in the tunneling current monitored during this procedure indicated a transition in the bilayer, as verified by subsequent imaging at 20 mV. The uncertainty of the angles determined from STM topographs is estimated to be ±3°.

### Computational Details

*Ab initio* calculations were performed with the DFT using the VASP code.^42^ Core electrons were treated using the projector augmented-wave method,^43^ and wave functions were expanded using a plane wave basis set with an energy cutoff of 500 eV. The PBE exchange and correlation functional^44^ was used in combination with the D3 method^45,46^ to treat van der Waals interactions.

The Pb(001) surface was simulated using the slab method with five layers of Pb and the experimentally determined $\left(\sqrt{17}\times \sqrt{17}\right)$ *R*14° in-plane unit cell, accommodating two molecules. A (3 × 3 × 1) *k* grid was used to sample the Brillouin zone. The positions of all atoms except the two bottom Pb layers were relaxed until all forces were smaller than 0.01 eV/Å.

Energy barriers were estimated using the climbing image nudge elastic band method (NEB).^47,48^ Charge transfers and spin magnetic moments were computed using the Bader approach.^49^ STM images were simulated following the Tersoff–Hamann approximation^50^ using the method of Bocquet et al.,^51^ as implemented in the STMpw code.^52^ Atomic plots were generated using the VESTA program.^53^

## Supplementary Material



## Notes

Different simulated configurations of the bilayer, adsorption energies and spin moments, projected density of states and spin densities, additional YSR results, and an image of a bilayer with some uncovered first layer (PDF)
