# Supplementary material for: Bilayer of Indium Phthalocyanine Molecules on Pb(001): Structure and Spin States
Source: J Phys Chem Lett. 2026 Aug 27;17(36):10362–9. doi: 10.1021/acs.jpclett.6c02063 (PMC13573617; doi:10.1021/acs.jpclett.6c02063)
Supplement: Supplementary file 1 [file jz6c02063_si_001.pdf]

Supporting Information for: Bilayer of Indium-Phthalocyanine Molecules on Pb(001): Structure and Spin States

Niklas Ide,[†] Arnab Banerjee,[†] Alexander Weismann,[†]
Roberto Robles,[*,‡] Nicolás Lorente,[‡,¶] and Richard Berndt[*,†]

†*Institut für Experimentelle und Angewandte Physik, Christian-Albrechts-Universität, 24098 Kiel, Germany*

‡*Centro de Física de Materiales CFM/MPC (CSIC-UPV/EHU), 20018 Donostia-San Sebastián, Spain*

¶*Donostia International Physics Center (DIPC), 20018 Donostia-San Sebastián, Spain*

* E-mail: roberto.robles@csic.es

* E-mail: berndt@physik.uni-kiel.de

Additional Theoretical Results

Figures S1 and S2 show different calculated structures of the InPc bilayer. We explored different adsorption sites and orientations for In↑Pc and In↓Pc. First, the images at high voltage ($V = 1.0\,\mathrm{V}$) show little variation and are therefore not useful to determine the correct structure. The images at low voltage (50 mV) show much more variation. The effect of molecular rotation can be seen by comparing structures I and II or structures VI and VII. The distinctive "doughnut" shape that appears in the experimental images requires a particular overlap of the molecules in first and second layers. A rotation can completely

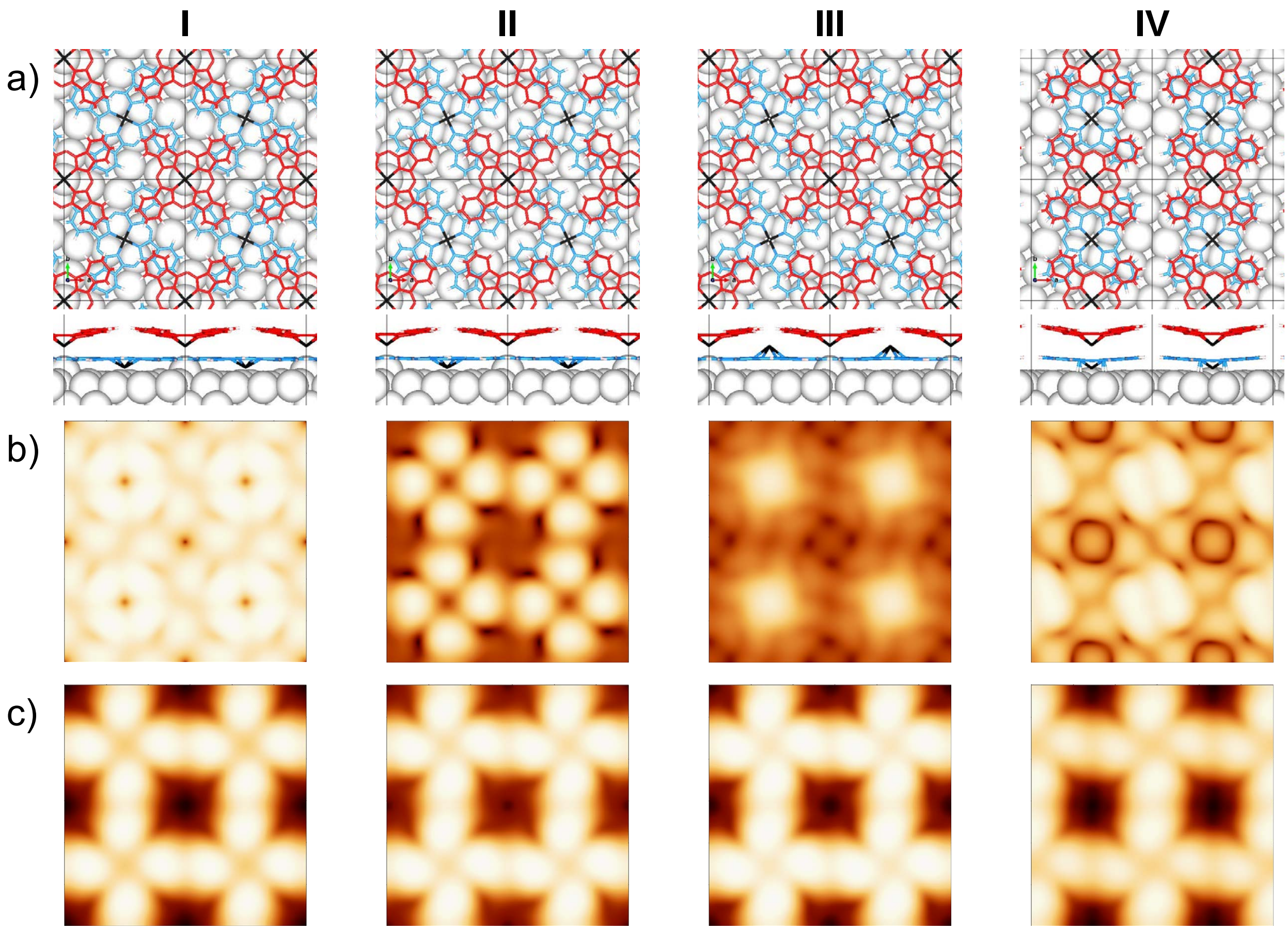

Figure S1: Each column shows data for a different solution of a bilayer on Pb(001). (a) Top and side views of the geometry. Molecules in the first (second) layer are represented by blue (red) sticks. White spheres represent Pb atoms. Black lines show the unit cell. Simulated constant-current STM images of the structures in (a) are shown for $V = 50\,\mathrm{mV}$ in (b) and $V = 1.0\,\mathrm{V}$ in (c). The area shown corresponds to (2×2) unit cells.

alter this shape. The effect of changing the lower molecule from In↓Pc to In↑Pc can be inspected by comparing structures VI and VIII. The images look quite similar except for the disappearance of the hole of the doughnut for In↑Pc in VIII. This pattern without a hole has never been observed in experiments. As can be seen by comparing V and VI, the adsorption site usually has a smaller influence on the simulated STM images.

We primarily examined cases in which the top molecule is positioned above the space between four first-layer molecules. Structure IV is an exception; in this structure, the top molecule is located between two bottom molecules. The resulting image is actually similar, but has C_2 symmetry instead of the C_4 symmetry. Interestingly, C_2 structures were observed

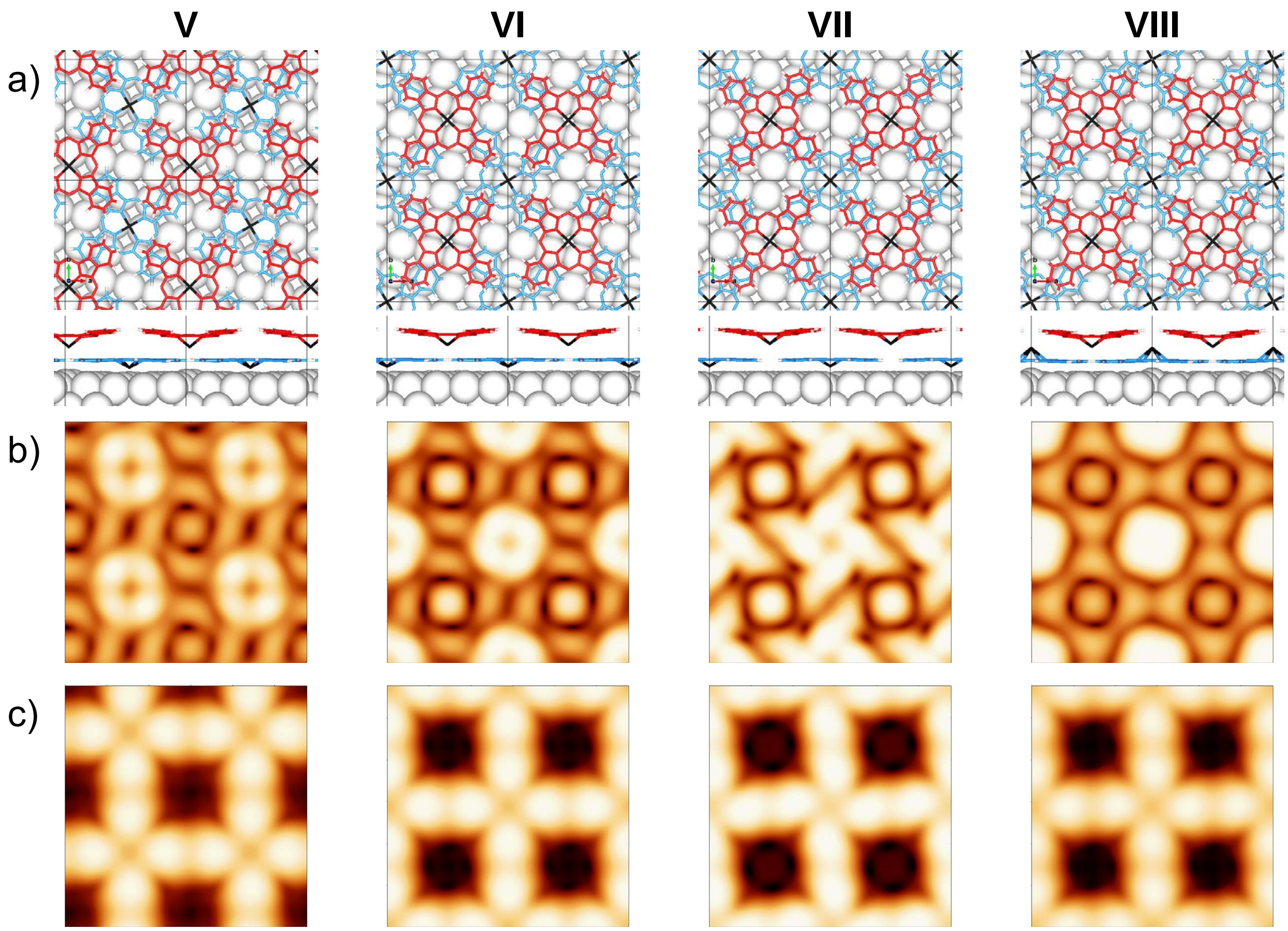

Figure S2: Each column shows data for a different solution of a bilayer on Pb(001). (a) Top and side views of the geometry. Molecules in the first (second) layer are represented by blue (red) sticks. White spheres represent Pb atoms. Black lines show the unit cell. Simulated constant-current STM images of the structures in (a) are shown for $V = 50\,\mathrm{mV}$ in (b) and $V = 1.0\,\mathrm{V}$ in (c). The area shown corresponds to (2×2) unit cells.

in the experiments. According to the results in Figures S1 and S2, solutions I, V, and VI may be compatible with the experimental results. However, among them only solution VI is magnetic (see Table SI), so we used it in the main text. It is worth noting that I is the energetically most stable solution (Table SI). DFT is known to fail to correctly describe the magnetic state of adsorbed molecules due to its inability to accurately account for the electronic correlation of the relevant molecular orbitals.[1] Therefore, structure I could be magnetic and correspond to the experimentally observed solution. In any case, however, the particular details of the structure do not affect the main results of this study.

Figure S3 shows the projected density of states (PDOS) for structure VI. The state at

Table SI: Adsorption energy E_{ad} (in eV per molecule) and spin moment μ (in μ_B) for the different solutions in Figures S1 and S2. Adsorption energies are defined as $E_{\rm ad} = [2 \cdot E[\rm InPc] + E[\rm Pb(001)] - E[\rm bilayer@Pb(001)]/2$, where E[InPc], E[Pb(001)], and E[bilayer@Pb(001)] are the energies of the gas-phase molecule, the Pb(001) surface, and the whole system, respectively. Mol 1 (2) denotes molecules at the first (second) layer.

	I	II	III	IV	V	VI	VII	VIII
$E_{\rm ad}$ (eV/mol)	3.217	3.175	2.812	2.928	2.956	2.882	2.852	2.647
μ[Mol 1] (μ_B)	0.00	0.00	0.00	0.00	0.00	-0.20	-0.06	-0.02
μ[Mol 2] (μ_B)	0.00	0.00	0.00	0.00	0.00	0.46	0.64	-0.56

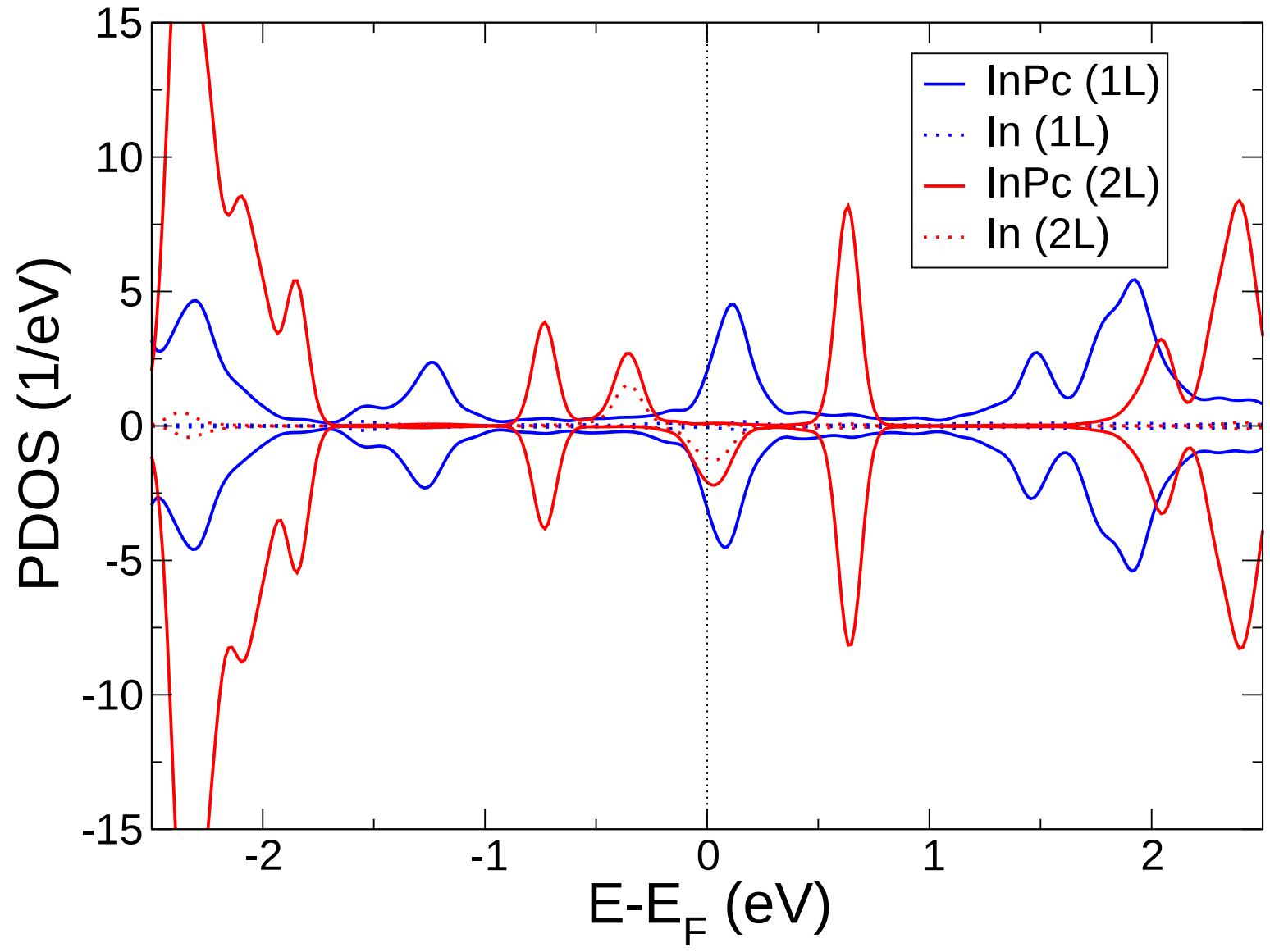

Figure S3: Projected density of states corresponding to structure VI. Solid blue (red) lines correspond to the molecule in the 1st (2nd) layer. Dashed lines correspond to states from the In atoms.

0.634 eV, which corresponds to the molecules on the 2nd layer, dominates the topographic STM images for voltages above this value. This produces the pattern in Figures S1c and S2c. A peak at a similar energy is present in all cases.

Figure S4 shows the spin density of structure VI. For molecules in the first layer, the spin density primarily concentrated within the tetrabenzo-tetraaza-porphyrin ring. For molecules in the second layer, the spin density is hight at the In ion and the nearby pyrrole N atoms.

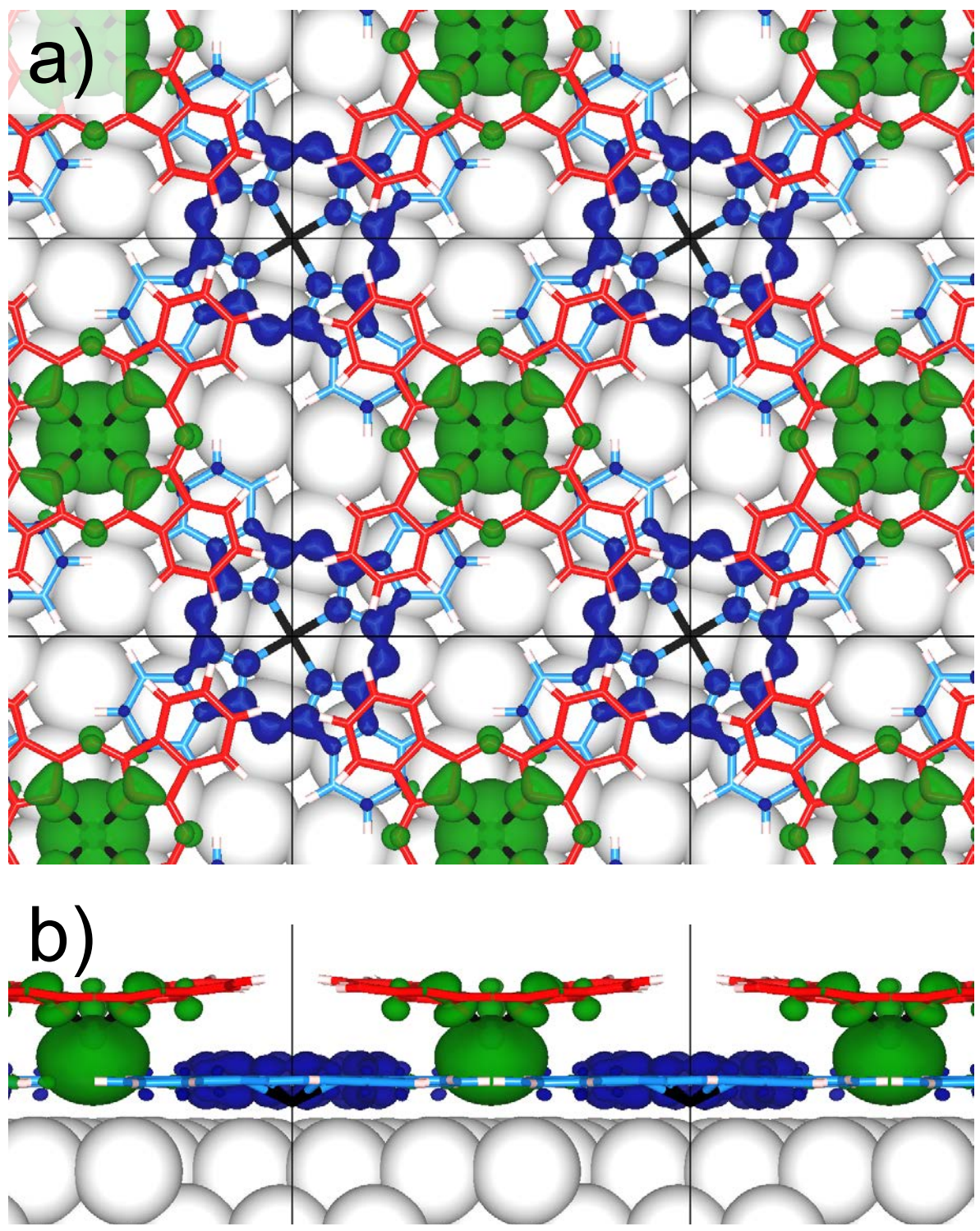

Figure S4: Spin density corresponding to structure VI. Green (blue) colors correspond to spin up (down) densities.

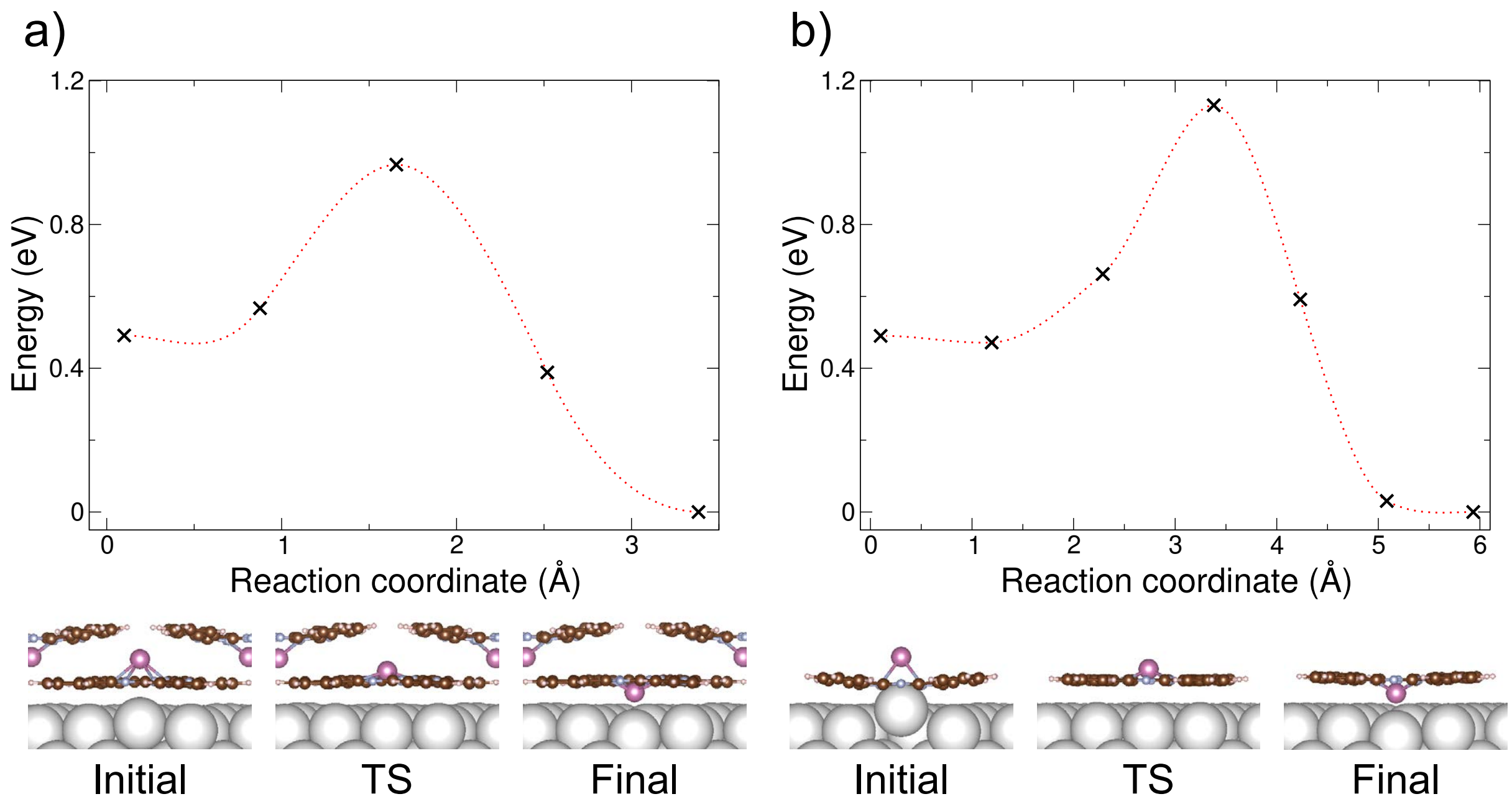

Figure S5: Energy barrier for the In↑Pc to In↓Pc transition for (a) the bilayer and (b) a monolayer using the same unit cell. The red lines are guides to the eye. At the bottom, ball-and-stick structures of the initial, final, and transition states (TS) are shown.

We calculated the energy barriers for the transition from In↑Pc to In↓Pc using the climbing image nudge elastic band method.[2,3] For the bilayer, the barrier is 0.475 eV. Using the same unit cell for a monolayer with the same arrangement, increases the barrier to 0.642 eV. Notably, the barrier is asymmetric; transitioning from In↓Pc to In↑Pc requires 0.967 eV for the bilayer and 1.132 eV for the monolayer.

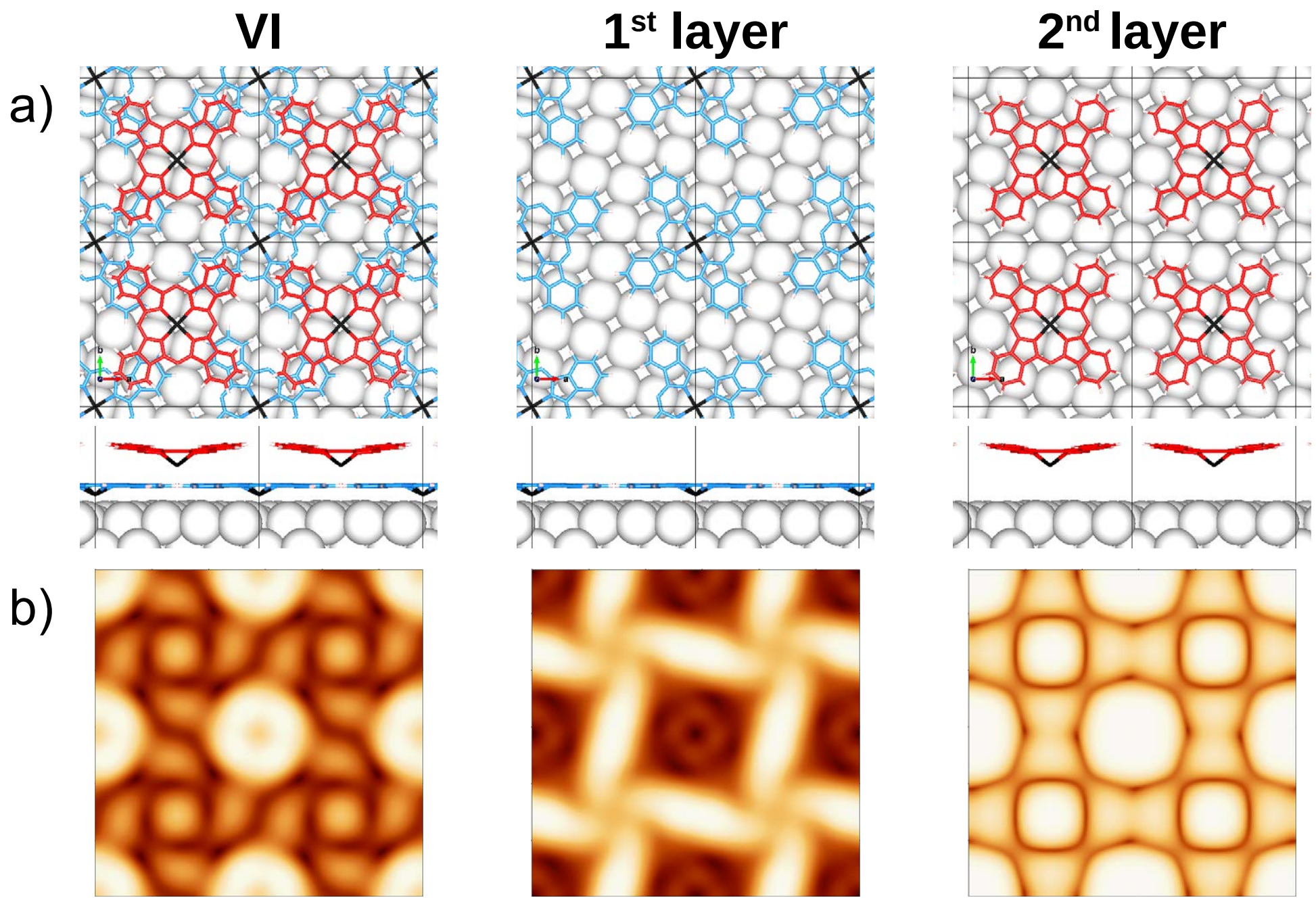

Figure S6: Left column correspond to structure VI, while in the other columns the same geometry is kept frozen, but keeping just the first (second) layer in central (right) columns. (a) Top and side views of the geometry. Molecules in the first (second) layer are represented by blue (red) sticks. White spheres represent Pb atoms. Black lines show the unit cell. Simulated constant-current STM images of the structures in (a) are shown for $V = 100\,\mathrm{mV}$ in (b). The area shown corresponds to (2×2) unit cells.

Figure S6 shows a set of calculations where starting from structure VI we maintained the geometry frozen, but kept only molecules in the first or second layer (Figure S6a). The simulated constant-current STM images (Figure S6b) show that the image for the full system resembles the image coming from the second layer, but modulated by the image from the first layer.

Additional Experimental Results

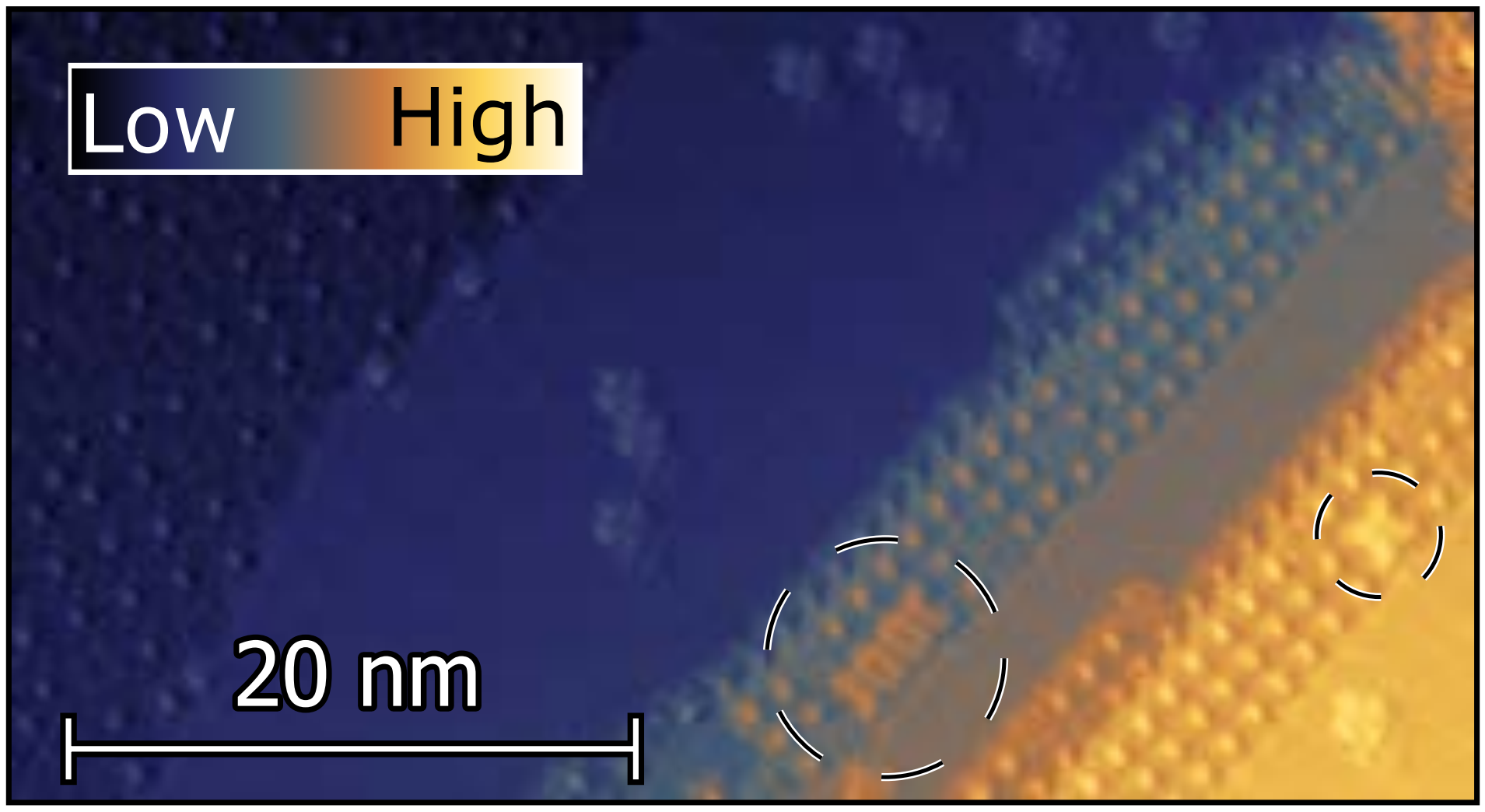

Figure S7: Topograph ($V = 1\,\mathrm{V}$) of a bilayer that does not completely cover the first layer. Circles indicate molecules in the second layer that grow on top of the first layer.

References

1. Mugarza, A.; Robles, R.; Krull, C.; Korytár, R.; Lorente, N.; Gambardella, P. Electronic and Magnetic Properties of Molecule-Metal Interfaces: Transition-Metal Phthalocyanines Adsorbed on Ag(100). *Phys. Rev. B* **2012**, *85*, 155437.

2. Henkelman, G.; Jónsson, H. Improved Tangent Estimate in the Nudged Elastic Band Method for Finding Minimum Energy Paths and Saddle Points. *J. Chem. Phys.* **2000**, *113*, 9978–9985.

3. Henkelman, G.; Uberuaga, B. P.; Jónsson, H. A Climbing Image Nudged Elastic Band Method for Finding Saddle Points and Minimum Energy Paths. *J. Chem. Phys.* **2000**, *113*, 9901–9904.
